# Supplementary material for: Circularization of 23S rRNA but not 16S rRNA within archaeal ribosomes
Source: Genome Biol. 2026 Jan 27;27:42. doi: 10.1186/s13059-025-03903-0 (PMC12918454; doi:10.1186/s13059-025-03903-0)
Supplement: Supplementary file 1 — Additional file 1: Figures S1-S16. Supplementary information of the paper [file 13059_2025_3903_MOESM1_ESM.pdf]

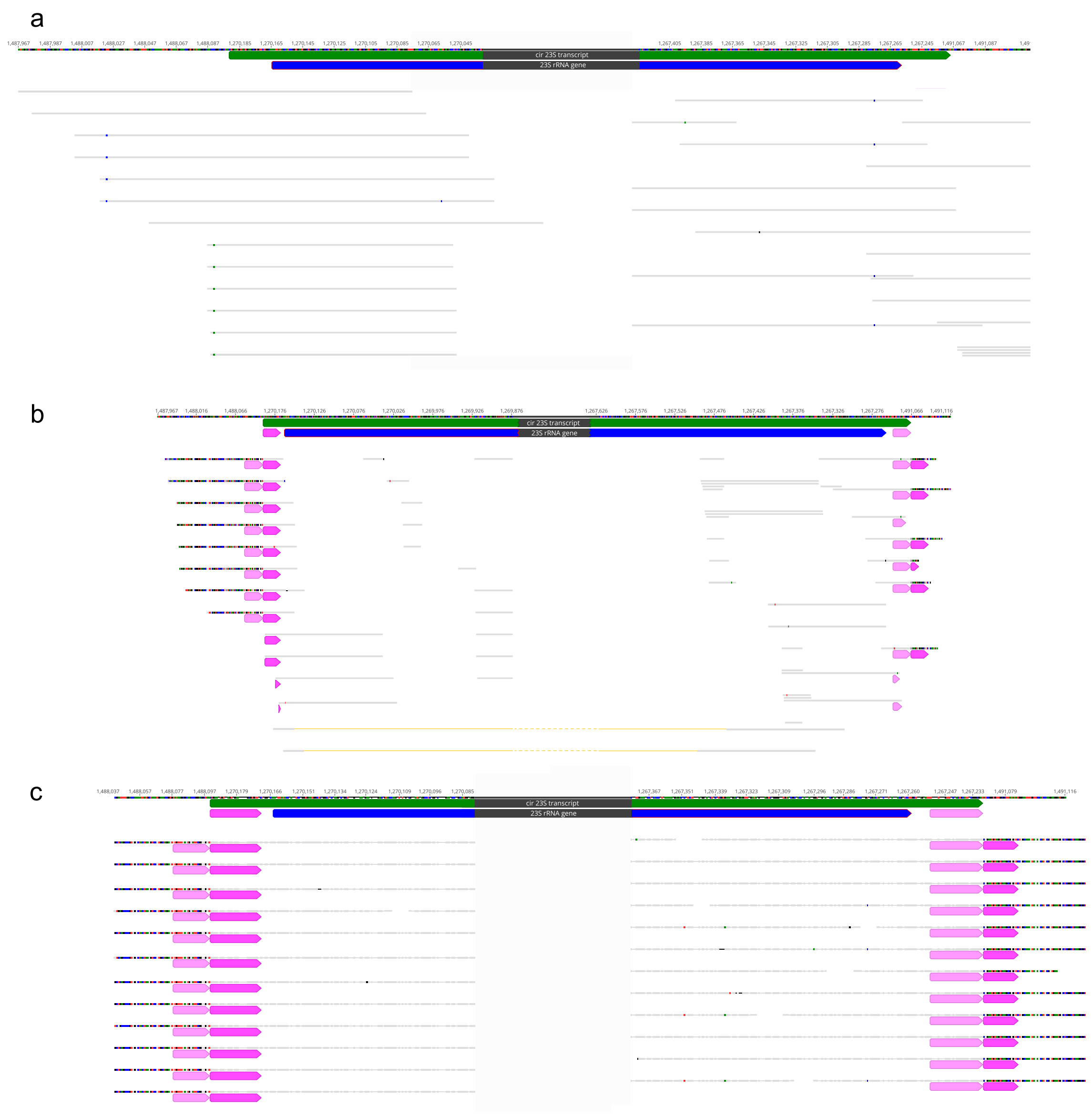

Fig. S1 Example of mapping of DNA sequences and RNA transcripts to the reference *Methanoperedens* genome. (a) Mapping using Illumina metagenomic DNA reads. (b) Mapping using Illumina metatranscriptomic RNA transcripts. (c) Mapping using Nanopore RNA transcripts. The long blue arrow indicates the predicted 23S rRNA gene in the genome. The green arrow shows the region of circular transcript inferred from transcript mapping. Gray bars are regions of mapped reads where the read sequence matches the reference; colored dots within reads indicate bases that do not agree with the reference. Colored arrows underneath reads indicate blocks that share the same sequence (e.g., the region that disagrees with the start of the circular transcript agrees with the region within the end of the circular transcript, and vice versa).

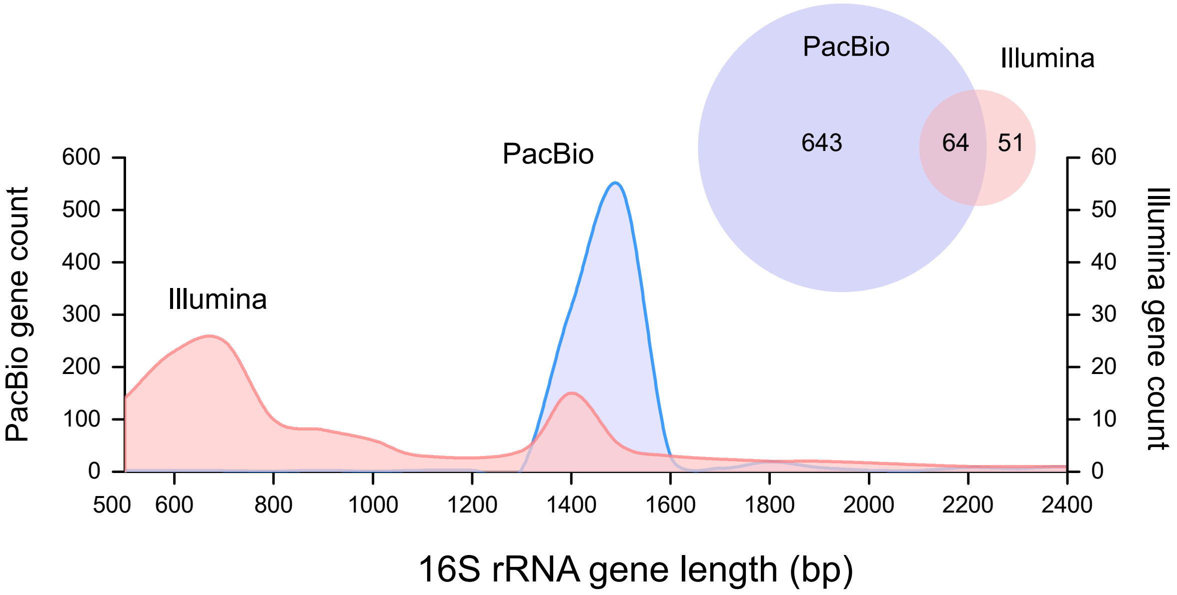

Fig. S2 Example of 16S rRNA genes assembled from PacBio and Illumina sequencing using the same soil sample (SR-VP_9_9_2021_81_5A_0_75m). The total data sizes are 38.6 Gb and 36.2 Gb, respectively, for PacBio and Illumina PE250 sequencing. The Venn diagram indicates the counts of distinct and shared 16S rRNA genes clustered at 99% of identity.

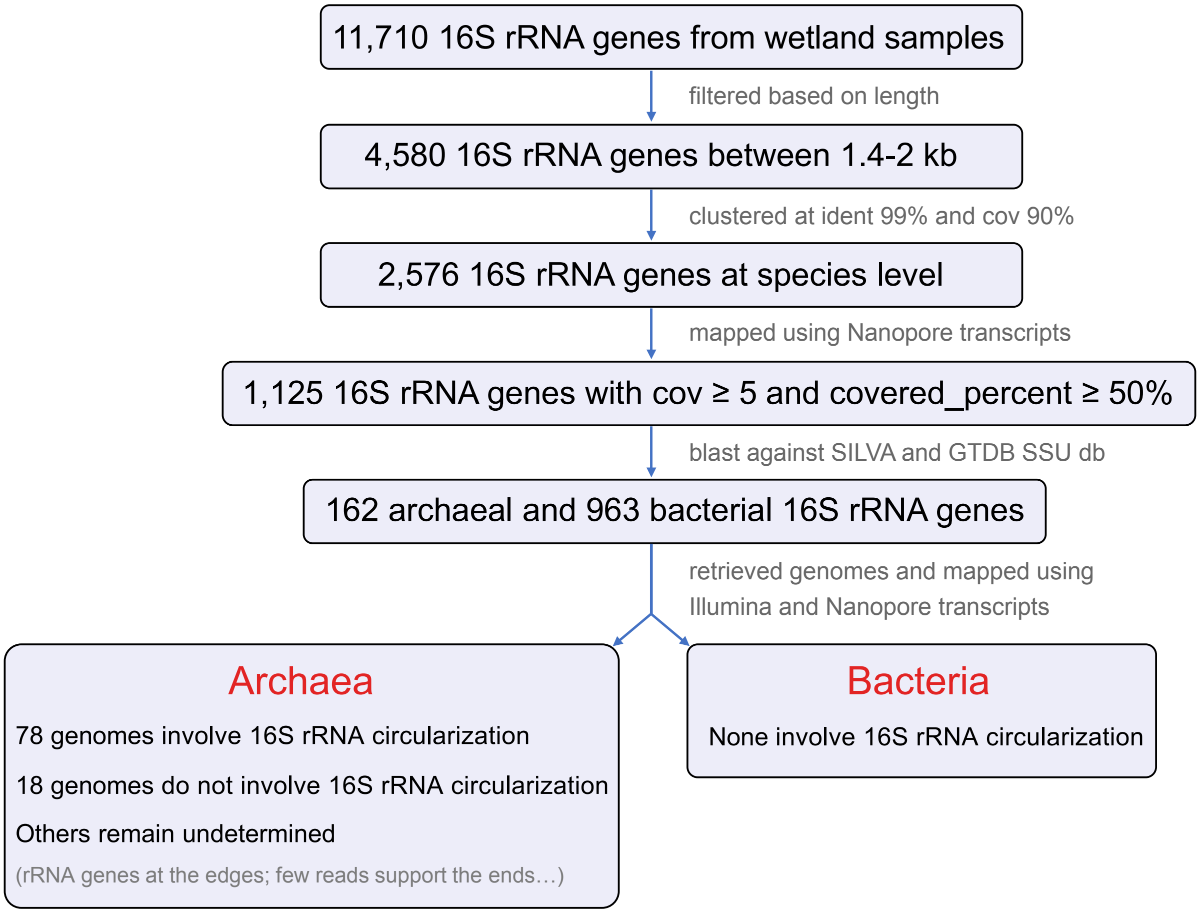

Fig. S3 Workflow of exploring 16S rRNA forms.

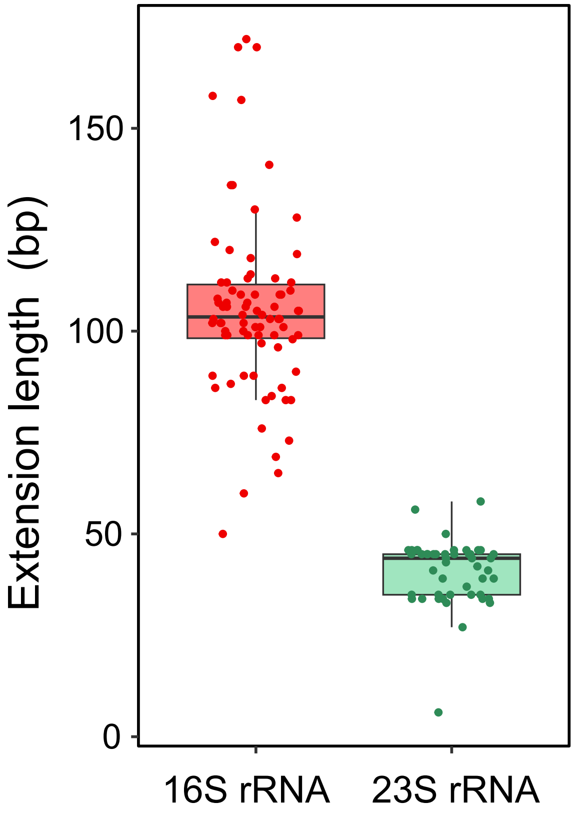

Fig. S4 Extension length of circular rRNA transcripts relative to genes predicted *in silico*. Colored dots indicate circular transcripts that contain 16S (n=78) and 23S rRNAs (n=48). Box plots show lower and upper quartiles and median values in each transcript group.

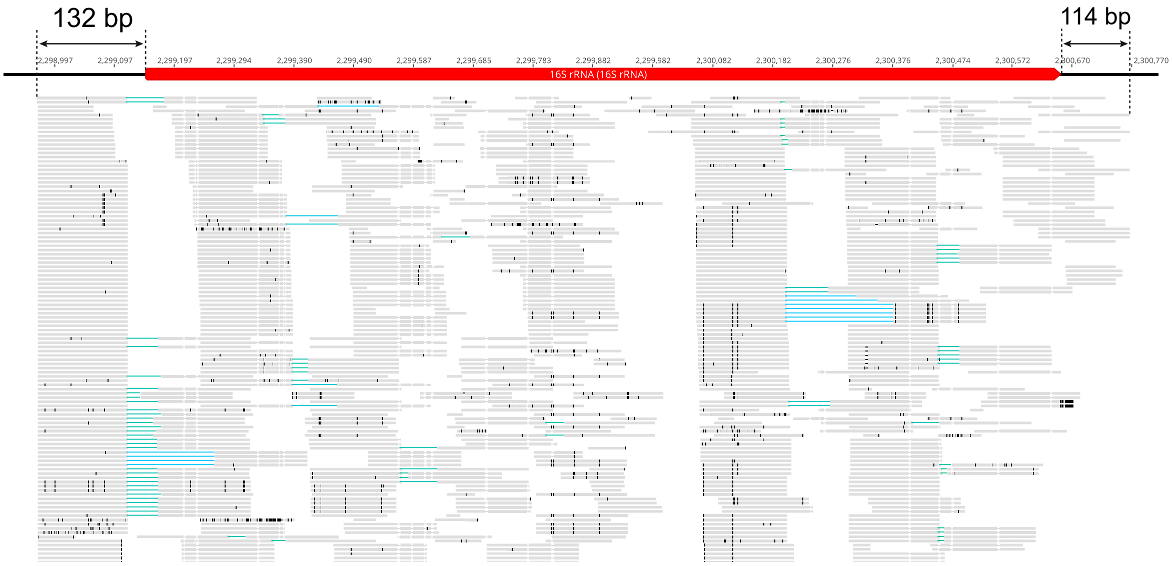

Fig. S5 Example of linear 16S rRNA transcript. This is a Bathyarchaeia genome fragment mapped by Illumina transcripts with maximum mismatches of 3%. The long red arrow in the genome reference indicates the predicted 16S rRNA gene. Gray bars are Illumina transcripts matching the reference and vertical black lines indicate mismatches to the reference. Green and blue indicate expected and larger than expected paired read separation distances.

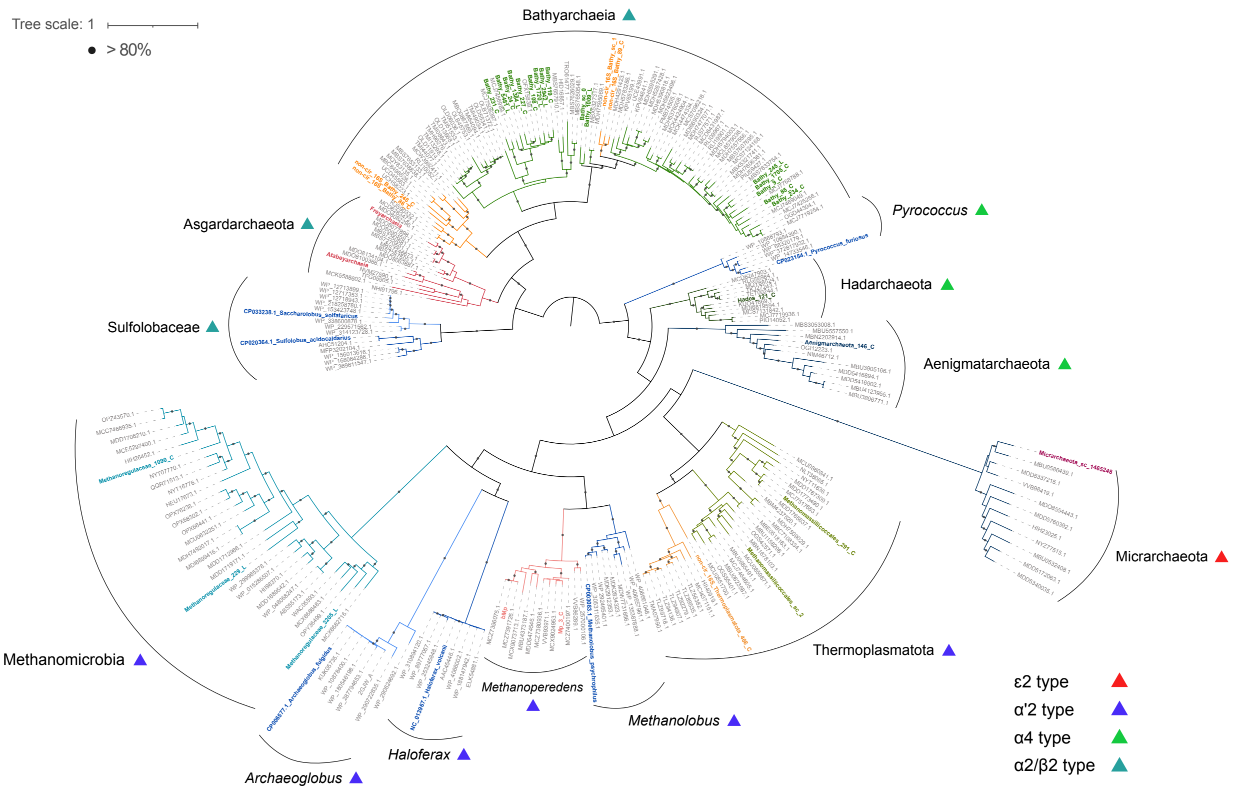

Fig. S6 Phylogeny of RNA splicing endonuclease (EndA). Sequences identified in our potentially complete or near-complete genomes are colored and in bold, except for the purple ones from references that can circularize 16S and 23S rRNAs. Colored triangles next to taxon names indicate different types of EndA. Genome information is present in Fig. 2 and Additional file 2: Table S1. Support values were calculated based on 1000 replicates and labeled as greater than 80.

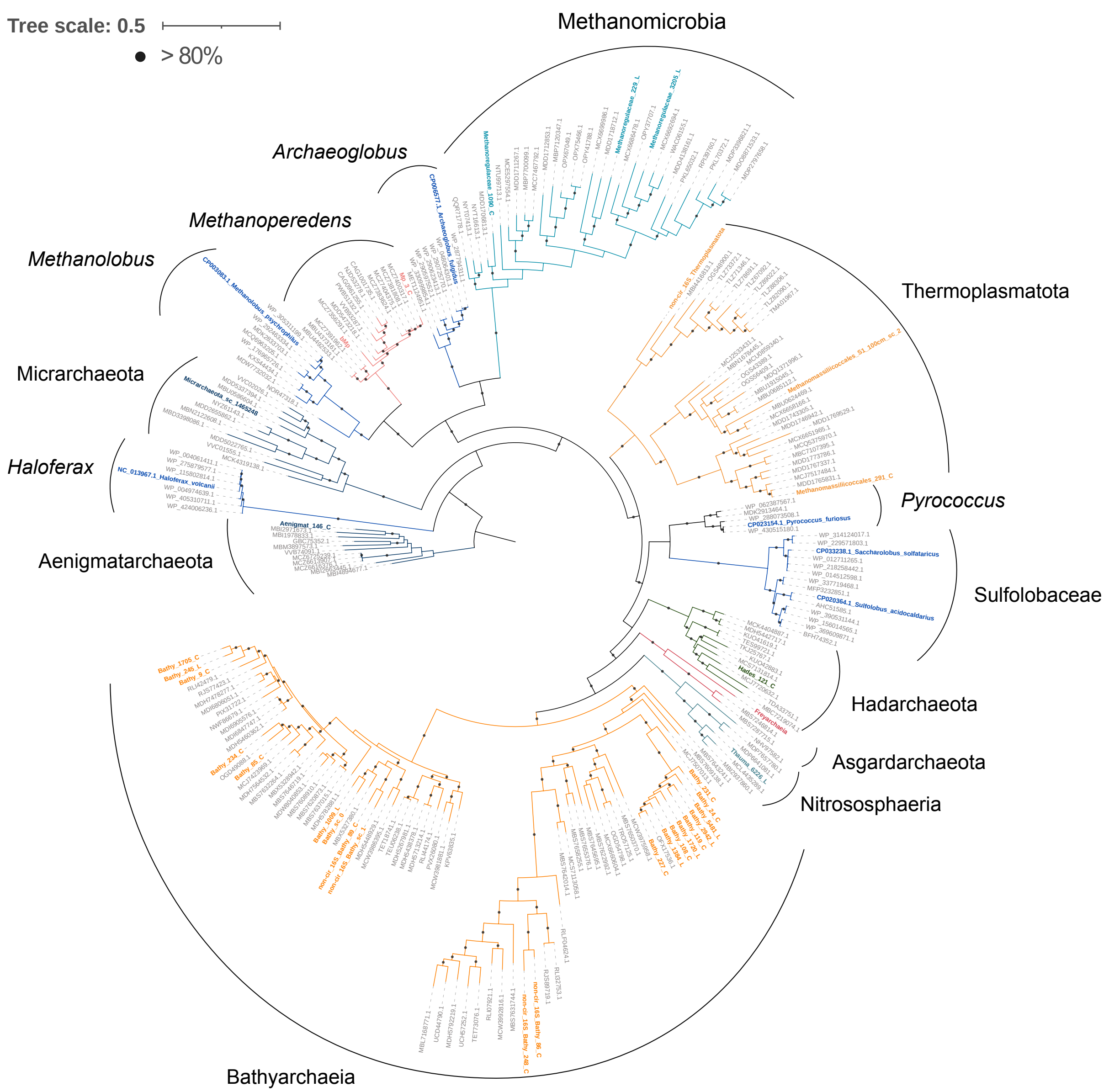

Fig. S7 Phylogeny of RNA ligase (RtcB). Sequences identified in our potentially complete or near-complete genomes are colored and in bold, except for the purple ones from references that can circularize 16S and 23S rRNAs. Genome information is present in Fig. 2 and Additional file 2: Table S1. Support values were calculated based on 1000 replicates and labeled as greater than 80.

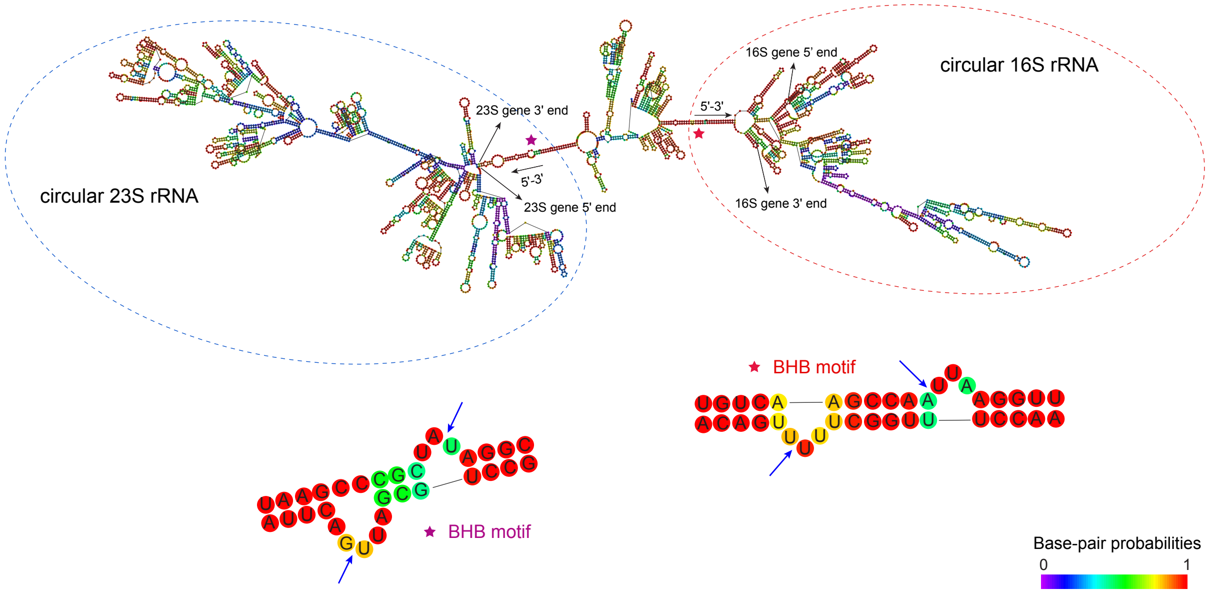

Fig. S8 Predicted secondary structure of the polycistronic transcript generating circular 16S and 23S rRNAs. The sequence is from a *Methanoperedens* genome as illustrated in Additional file 1: Fig. S1. BHB splicing motifs in the transcript are labeled with stars and detailed at the bottom. Blue arrows indicate the cleavage sites on the BHB motifs.

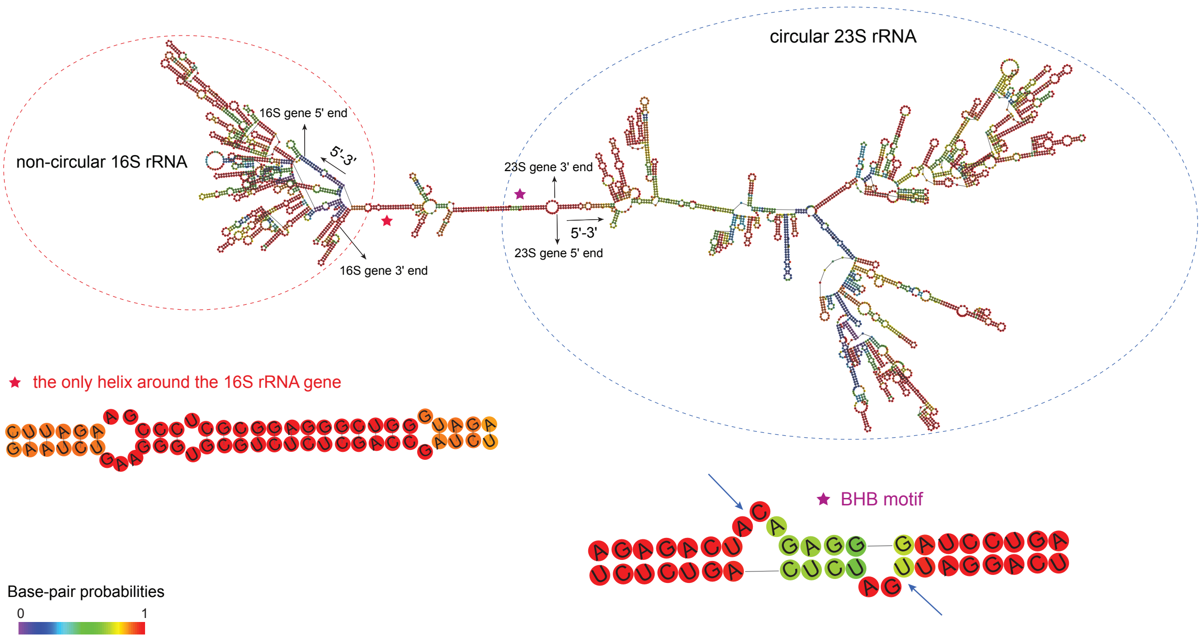

Fig. S9 Predicted secondary structure of the polycistronic transcript generating non-circular 16S and circular 23S rRNAs. The sequence is from a Bathyarchaeia genome as illustrated in Additional file 1: Fig. S5. The only helix around the 16S rRNA gene and the BHB motif around the 23S rRNA gene are labeled with stars and detailed at the bottom. The blue arrow indicates the cleavage sites on the BHB motif.

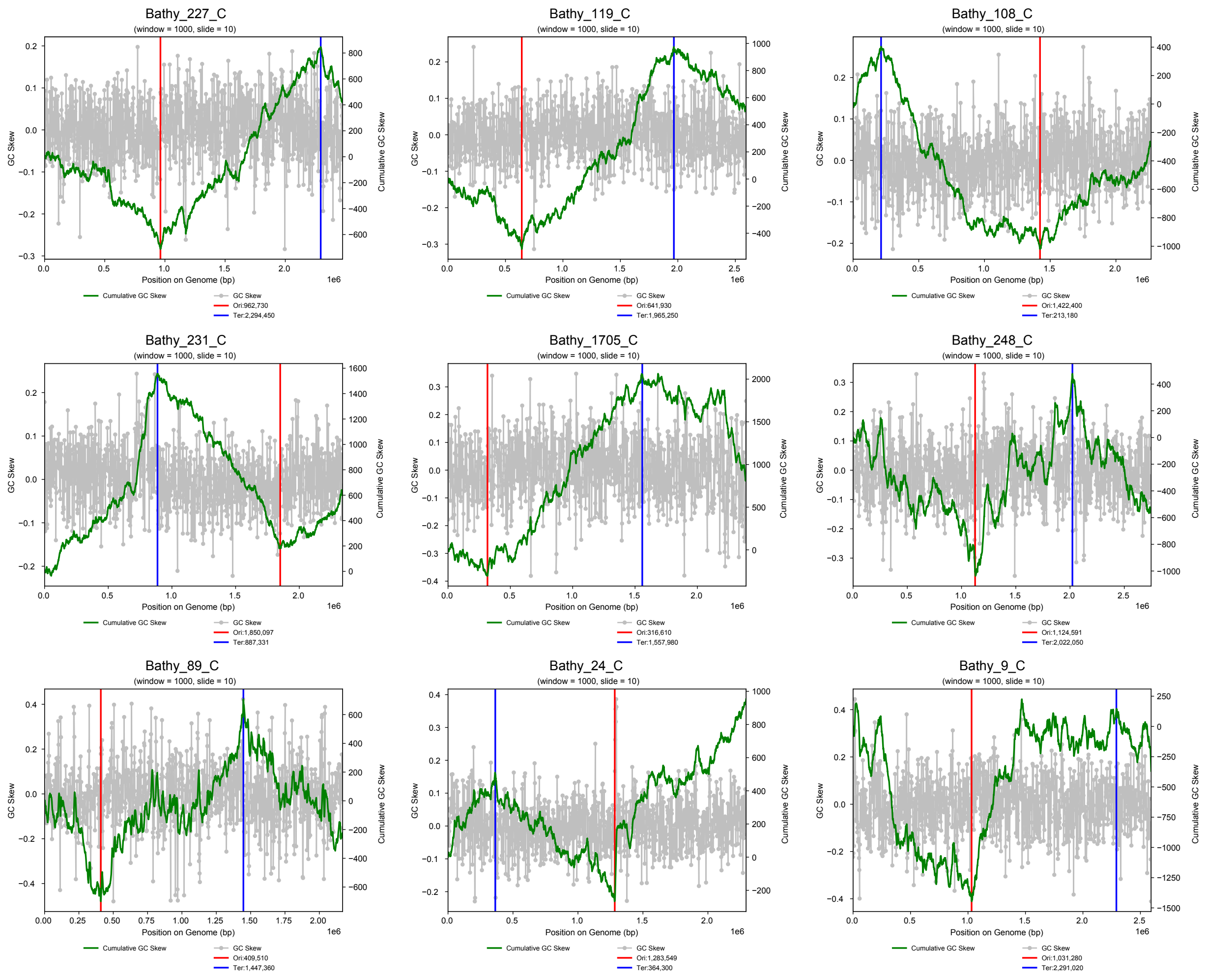

Fig. S10 GC skew profiles of circular Bathyarchaeia genomes. Gray dots and green lines indicate GC skew and cumulative GC skew across genomes. Red and blue lines show the inferred locations of replication origins and termini.

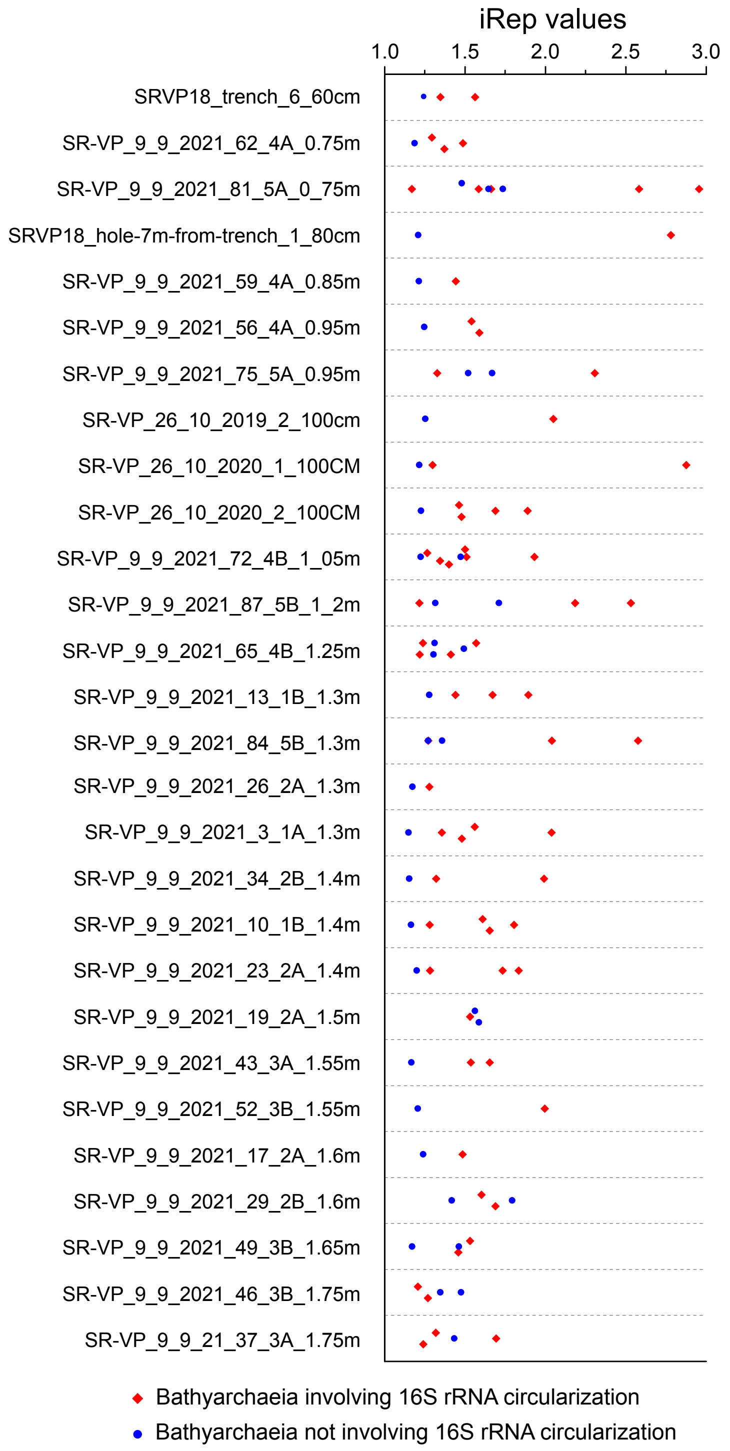

Fig. S11 *in situ* replication rates of Bathyarchaeia genomes generating circular or non-circular 16S rRNA intermediates. Samples are shown only if both genome types co-existed there. Significant differences in iRep values are observed between the two genome types ($p=0.000015$), using the Wilcoxon signed-rank test.

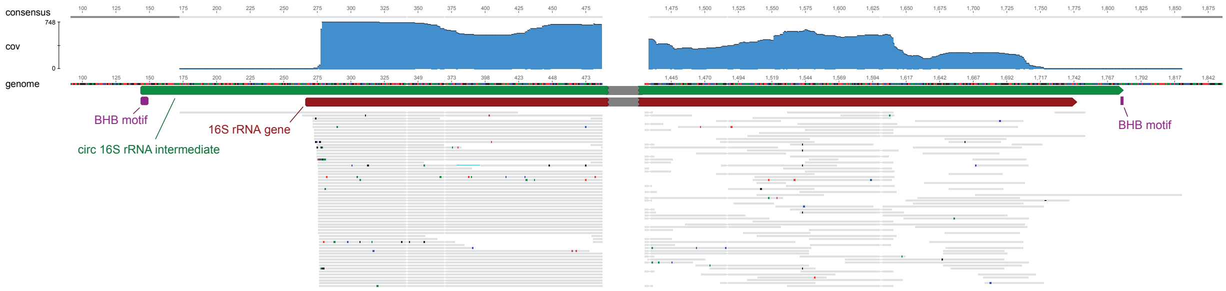

Fig. S12 Linear 16S rRNA within the *Methanosarcina acetivorans* ribosome. *M. acetivorans* genome was mapped by 250-bp transcript reads. The dark red arrow indicates the 16S rRNA gene predicted by Rfam, and the green arrow indicates its circular transcript intermediate inferred from transcript read mapping. Purple boxes show the bulge regions in the BHB splicing motif. Gray bars are mapped reads, in which colored dots are mismatched nucleotides to the genome. No reads support the circularization of 16S rRNA within the *M. acetivorans* ribosomes.

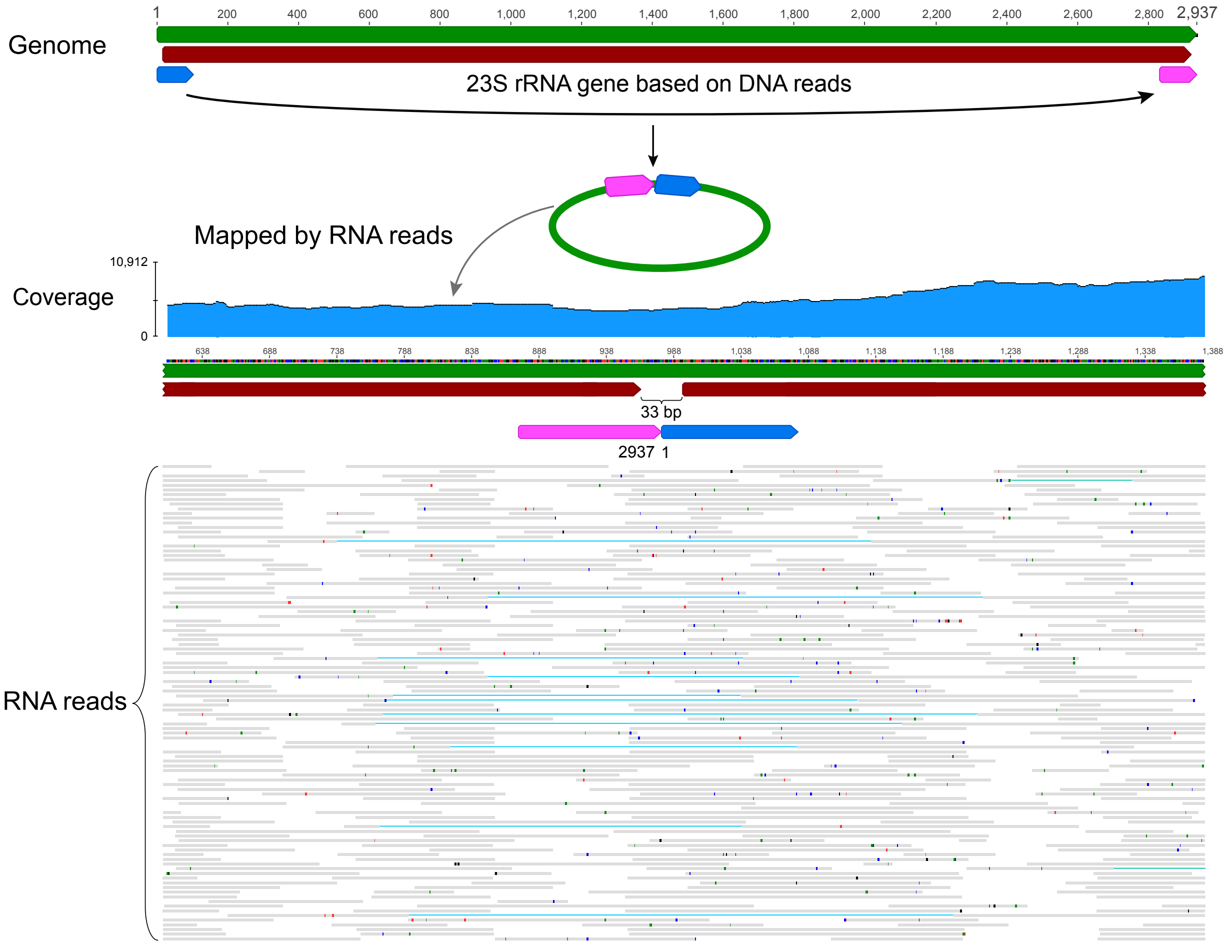

Fig. S13 Transcript mapping of circular 23S rRNA within the *Methanosarcina acetivorans* ribosome. The blue and pink arrows correspond to those in Fig. 4a. The region labeled with arrows in the permuted circular 23S rRNA is mapped with RNA reads. Gray bars show mapped transcript reads wherein colored dots are mismatched nucleotides to the reference.

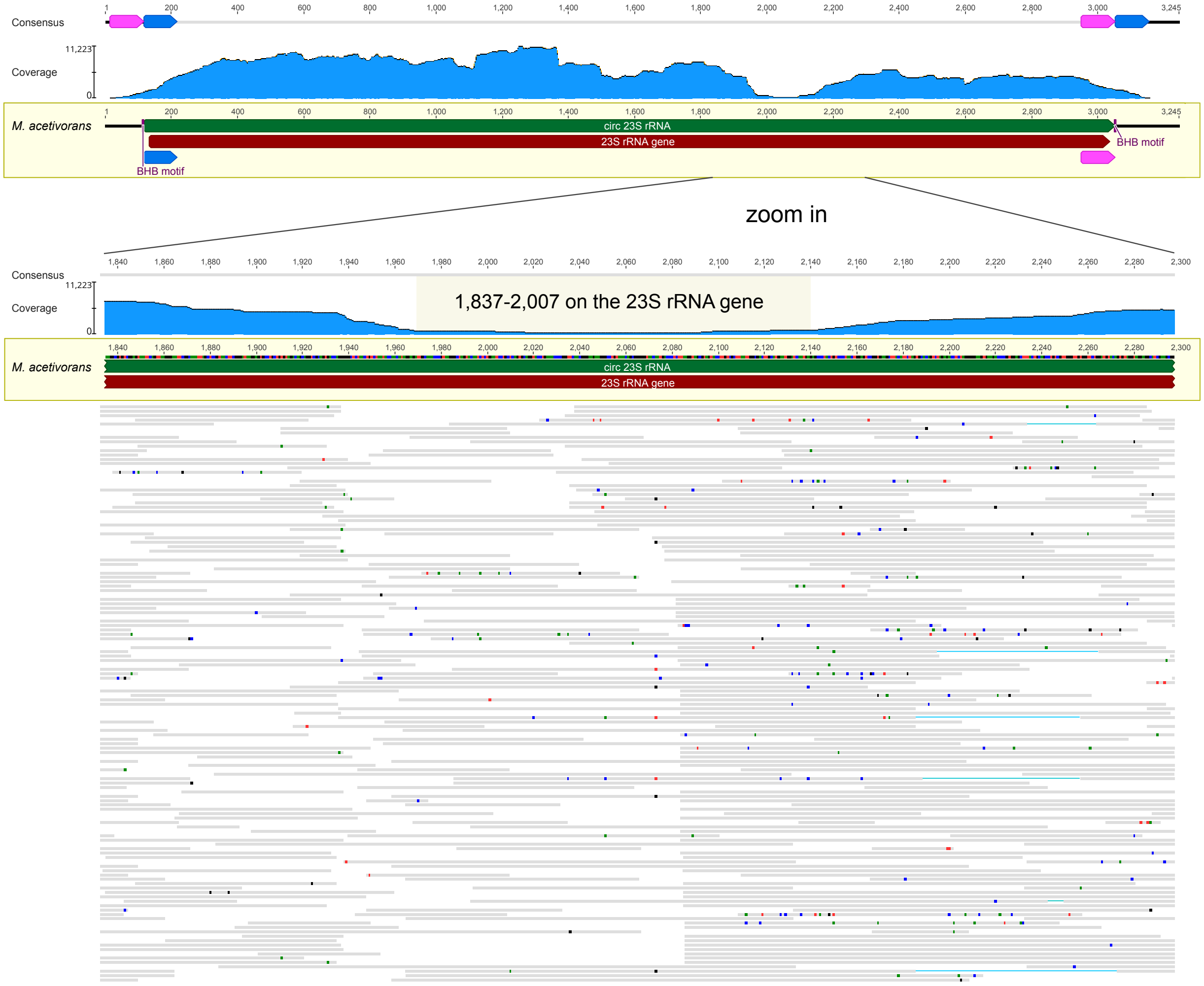

Fig. S14 Transcript read mapping of the full-length 23S rRNA gene of *M. acetivorans*. Gray bars are mapped reads, in which colored dots indicate nucleotides that do not match the reference sequence. The only coverage "dip" is located at positions 1,837-2,007 of the 23S rRNA gene, which is predicted to be domain IV (Fig. 4c). Detailed mapping of the gene edges is present in Fig. 4a.

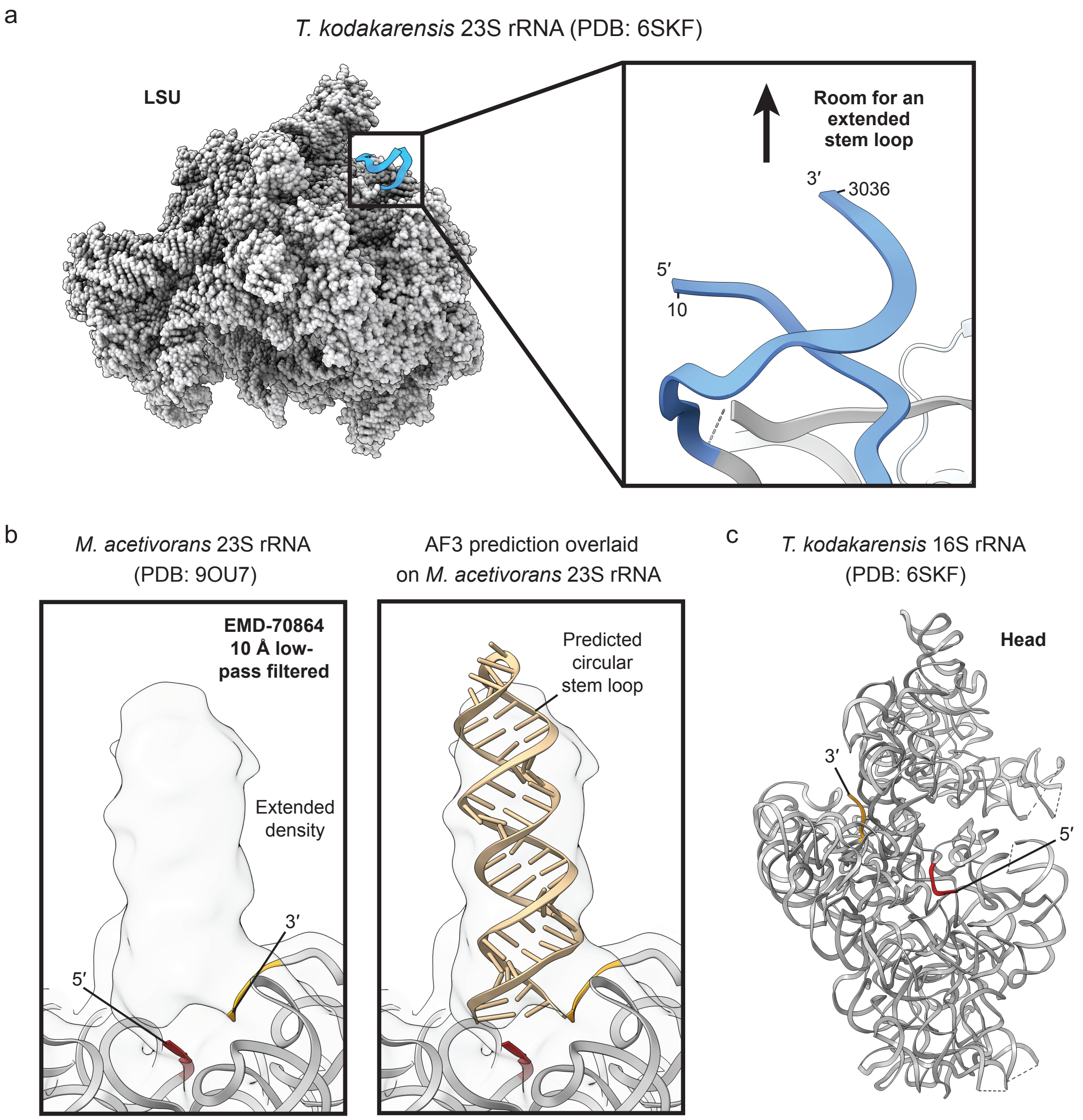

Fig. S15 Structural analysis of archaeal ribosomes. (**a**) The 5′ and 3′ ends of the *Thermococcus kodakarensis* 23S rRNA are shown in blue. The residue numbers where the 23S rRNA model begins and ends in PDB 6SKF are indicated. (**b**) In the left panel, the 5′ (red) and 3′ (orange) ends of *M. acetivorans* 23S rRNA (PDB: 9OU7) are overlaid on a 10 Å low-pass filtered cryo-EM map of the *M. acetivorans* large subunit (EMD-70864). Cryo-EM density can be seen extending away from the surface of the ribosome. In the right panel, an AlphaFold 3 prediction of the circularized 23S rRNA stem-loop is overlaid on the *M. acetivorans* 23S rRNA model and low-pass filtered cryo-EM map. (**c**) 5′ (red) and 3′ (orange) ends are highlighted in the *T. kodakarensis* 16S rRNA from PDB 6SKF.

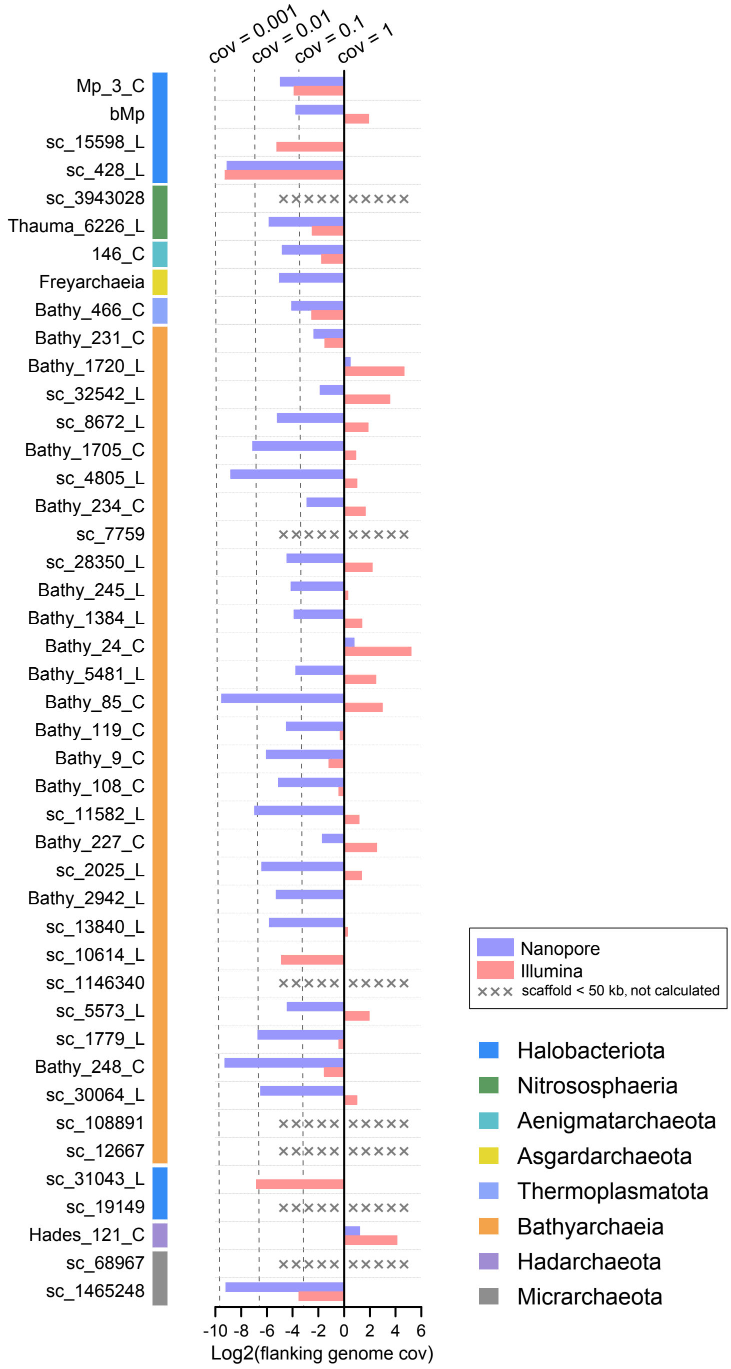

Fig. S16 Average transcript coverages of genomes flanking rRNA genes. Both Nanopore and Illumina transcripts were mapped to flanking genomes with maximum mismatches of 3%. Average coverages were calculated by dividing total mapped transcript bases by flanking genome length and converted logarithmically with base 2. To avoid biases caused by too few genes, only genomes (scaffolds) ≥ 50 kb were included.
